# Investigating the In Vitro Regeneration Potential of Commercial Cultivars of *Brassica*

**DOI:** 10.3390/plants8120558

**Published:** 2019-11-29

**Authors:** Nisma Farooq, Muhammad Asif Nawaz, Zahid Mukhtar, Iftikhar Ali, Penny Hundleby, Niaz Ahmad

**Affiliations:** 1 Agricultural Biotechnology Division, National Institute for Biotechnology and Genetic Engineering (NIBGE), Faisalabad 38000, Pakistan; 2 Nuclear Institute for Agriculture and Biology, Faisalabad 38000, Pakistan; 3 Department of Crop Genetics, John Innes Centre, Norwich Research Park, Norwich NR4 7UH, UK

**Keywords:** in vitro regeneration, tissue culture, commercial cultivars, *Brassica juncea*, *Brassica napus*

## Abstract

In vitro regeneration is a pre-requisite for developing transgenic plants through tissue culture-based genetic engineering approaches. Huge variations among different genotypes of the genus *Brassica* necessitate the identification of a set of regeneration conditions for a genotype, which can be reliably used in transformation experiments. In this study, we evaluated the morphogenesis potential of four commercial cultivars (Faisal canola, Punjab canola, Aari canola, Nifa Gold) and one model, Westar, from four different explants namely cotyledons, hypocotyls, petioles and roots on three different *Brassica* regeneration protocols, BRP-I, -II and -III. The regeneration efficiency was observed in the range of 6–73%, 4–79.3%, 0–50.6%, and 0–42.6% from cotyledons, petioles, hypocotyls and roots, respectively, whereas, the regeneration response in terms of average shoots per explant was found to be 0.76–10.9, 0.2–3.2, 0–3.4 and 0–2.7 from these explants. Of the commercial varieties tested, almost all varieties showed poorer regeneration than Westar except Aari canola. In comparison to Westar, its regeneration frequency from cotyledons was up to 7.5-fold higher on BRP-I, while it produced up to 21.9-fold more shoots per explant. Our data show that the explant has strong influence on the regeneration response, ranging from 24% to 92%. While the growth of commercial cultivars was least affected by the regeneration conditions provided, the effect on Westar was twice that of the commercial cultivars. After determining the optimal explant type and regeneration conditions, we also determined the minimum kanamycin concentration levels required to selectively inhibit the growth of untransformed cells for these cultivars. Regenerated shoots of Aari canola could be successfully grown to maturity within 16–18 weeks, with no altered phenotype noted and normal seed yields obtained. Therefore, the commercial variety, Aari canola, could be a good candidate for future genetic transformation studies.

## 1. Introduction

*Brassica*, from the family Brassicaceae, is an economically important genus. It includes several species that are often used as oilseed crops, vegetables, fodder crops as well as condiments. *Brassica* oilseed varieties producing oil low in anti-nutritive aliphatic glucosinolates and erucic acid as well as rich in unsaturated fatty acids are generally termed as ‘canola’. Conventionally, the term ‘canola’ was more often used for *B. napus* but now some canola quality varieties of *B. rapa* and *B. juncea* are also available [1,2,3,4]. Being rich in omega-6 and omega-3 fatty acids and low saturate fats, canola oil is considered as a heart-healthy oil. Due to its high quantity of proteins, its meal for poultry and livestock is considered as good as soybean [5,6].

With the increasing world population, the demand for vegetable oil is also increasing. According to the United States Department of Agriculture, the largest importers of vegetable oil in 2018 were the European Union, the US, China and India. Pakistan also gets over 80% of its edible oil requirements from imports [7]. Since the increase in cultivable land is not possible in the face of an increasing population, the viable option to meet the challenges of edible oil requirements, as well as industrial applications, is to develop stress-resilient high-yielding *Brassica* genotypes. The development of stress tolerant *Brassica* is possible by transferring genes from the plant species that are adapted to harsh environmental conditions. These species present a rich reservoir of the traits that enable them to grow under stressful conditions. However, transferring these traits to salt or drought sensitive crops is only possible by genetic transformation, as they cannot be cross bred through conventional breeding approaches. GM (genetic manipulation) tools developed in the 1980s allow the transferring of traits from a wide taxon for engineering novel traits into field crops. Herbicide tolerance, insect resistance, β-carotene synthesis (golden rice) and vitamin-enrichment (multivitamin corn) are few examples among the list of engineered traits through GM technology.

The introduction of transgenes into plants to engineer useful novel traits may seem trivial now [8], but it has its own challenges and limitations [9,10,11,12]. For example, a plant species must be responsive to in vitro regeneration protocols, and a robust regeneration system is one of the key pre-requisites for successful genetic transformation. Several indigenous *Brassica* varieties developed locally have canola characteristics. Being stress-sensitive, these varieties are unable to grow on marginal lands. To develop stress-resilient transgenic versions, it is necessary to determine the morphogenesis potential of these varieties. Although transformation of *Brassica* species has been reported in several studies [13,14,15,16,17,18,19,20,21,22], several *Brassica* genotypes remain recalcitrant to genetic transformation [2,19,23]. Several factors including susceptibility to *Agrobacterium* infection, choice of explant and tissue culture conditions mainly responsible for these variations have been identified [13,19,22]. These factors vary from genotype to genotype, indicating a strong genetic control on in vitro regeneration and transformation of *Brassica* genotypes [22,24,25,26]. Therefore, it is important to find out responsive genotypes, as well as a type of explant, which show reliable regeneration efficiency to be used in future transformation experiments.

Traditionally, model cultivars such as Westar have been used in transformation and regeneration experiments for the introduction of transgenes for desired traits [19,27]. While these model cultivars are valuable for studying gene function, there is a need to work directly on elite cultivars for the development of climate-resilient crops. The undesired agronomic characters presented by the model plants require an exhaustive process of crossing and back-crossing to transfer engineered traits into field varieties [10]. The challenge of transferring traits from model plants is greatly increased if the number of genes to introduce increases. Therefore, the transformation of commercial cultivars with desired agronomic performance adapted to the prevailing climatic conditions is highly desirable, making the process of developing transgenic plants quicker and more efficient. However, their transformation is often hampered with genotypic recalcitrance, which makes it necessary to test them for in vitro regeneration prior to genetic transformation experiments.

In this study, we evaluated the regeneration potential of different commercial varieties of *Brassica* grown in Pakistan using different explants and growth conditions for establishing transgenic technology in commercial varieties of *Brassica*. One of the commercial varieties, Aari canola, was found to be highly responsive to the given conditions. We also determined kanamycin concentrations to be used in future transformation experiments for the recovery of transformants. The in vitro Aari canola plants grown to maturity showed a normal plant morphology, with normal seed setting and without any observable phenotypic variations. The information generated in this study will be useful for developing stress-resilient *Brassica* varieties by directly transforming the commercial cultivars.

## 2. Results

### 2.1. Shoot Regeneration from Cotyledons

All the explants, including cotyledons, normally regenerated multiple shoots and occasionally a single shoot per explant. Regenerates formed from the cut end of the 2 mm petiole attached to the cotyledon. It was essential when explants were isolated that no traces of meristem region were left behind during the explant cutting to avoid the emergence of shoots from meristematic tissue (however, it is acknowledged that for those not familiar with the technique this can be difficult at first, but shoots resulting from meristematic tissue will be visible as shoots emerging within a few days of explant isolation and should be discarded). Typically, the explants would swell in the first week of incubation, and shoot primordia will start appearing in the second week. The regeneration efficiency was calculated as following:

Regeneration efficiency=No of explants showing regenerationtotal number of explants  × 100

**Figure 1:**
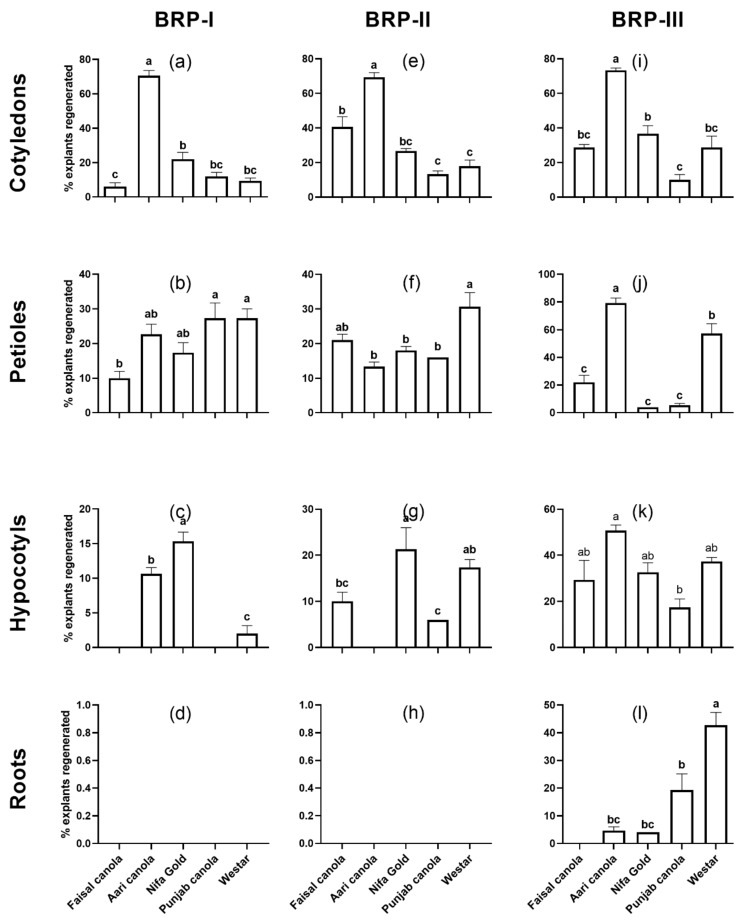
Regeneration efficiency of five *Brassica* cultivars from different explants on three different regeneration protocols, BRP-I (**a**–**d**), BRP-II (**e**–**h**) and BRP-III (**i**–**l**) from cotyledons (**a**,**e**,**i**), petioles (**b**,**f**,**j**), hypocotyls (**c**,**g**,**k**) and roots (**d**,**h**,**l**). Data points represent the mean ± SE of three replicates.

The highest regeneration efficiency was observed for Aari canola on all the three protocols with 70.6% on BRP-I, 69.3% on BRP-II, and 73.3% on BRP-III (Figure 1a,e,i). Faisal canola showed the lowest regeneration efficiency on BRP-I (6.0%) and it was Punjab canola which showed the lowest regeneration on BRP-II and BRP-III with 13.3% and 10.0% efficiency, respectively. The regeneration efficiency of Aari canola was found to be 7.5-fold higher than the model, Westar, on BRP-I, 3.8-fold higher on BRP-II and 2.5-fold higher on BRP-III.

**Figure 2:**
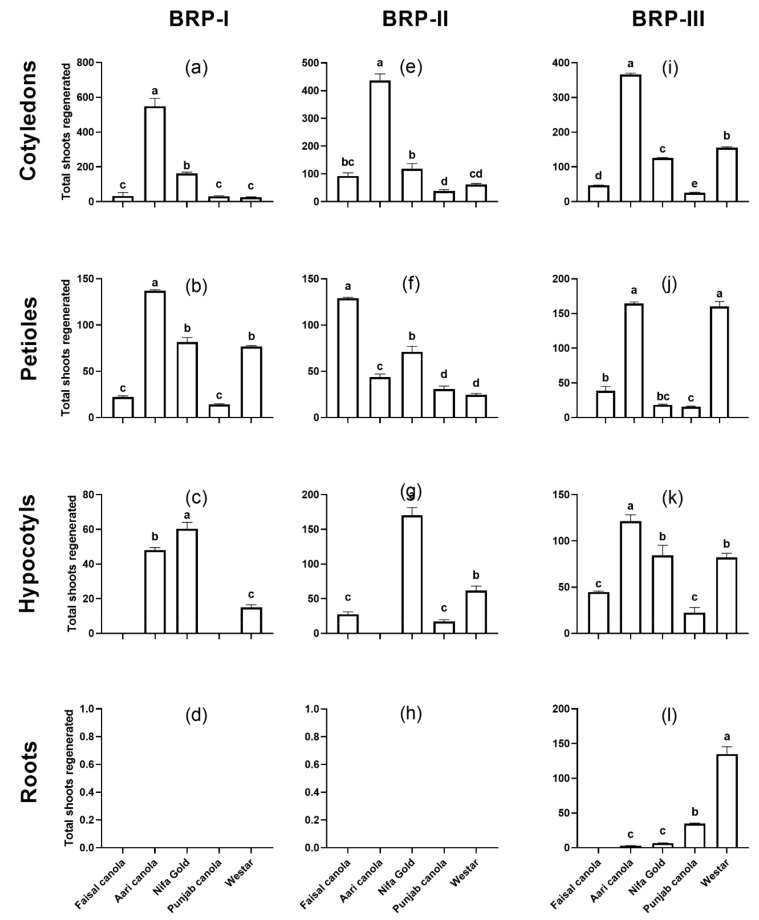
Regeneration of total number of shoots from different explants of five *Brassica* cultivars on three different regeneration protocols, BRP-I (**a**–**d**), BRP-II (**e**–**h**) and BRP-III (**i**–**l**) from cotyledons (**a**,**e**,**i**), petioles (**b**,**f**,**j**), hypocotyls (**c**,**g**,**k**) and roots (**d**,**h**,**l**). Data points represent the mean ± SE of three replicates.

In terms of total shoots regenerated, the highest shoot formation was observed in Aari canola on all the three protocols with 548 shoots on BRP-I (average 10.96 shoots/explant), 436.3 on BRP-II (8.72 shoots/explant) and 366.3 on BRP-III (7.3 shoots/explant) from a total of 50 explants (Figure 2a,e,i). The least regeneration responsive was Westar (25 shoots/50 explants; 0.50/explant) on BRP-I, and Punjab canola on BRP-II (38.3 shoots/50 explants; 0.76 shoots/explant) and BRP-III (25 shoots/50 explants; 0.50/explant). In terms of average number of shoots per explant, it was in the range of 0.50–10.9, 0.76–8.7 and 0.5–7.3 from cotyledons on BRP-I BRP-II and BRP-III, respectively. Overall, the regeneration response of Aari canola, in terms of total shoots formation, was 21.9-fold higher than Westar on BRP-I, 7.0-fold higher on BRP-II and 2.3-fold higher on BRP-III.

### 2.2. Shoot Regeneration from Detached Petioles

Petiole explants regenerated shoots along the middle rib after 3 weeks on SIM. The highest regeneration efficiency from detached petioles was observed for Aari canola on BRP-III (79.3%), Westar on BRP-I and BRP-II with a regeneration of 27.3% and 30.6%, respectively (Figure 1b,f,j). The lowest regeneration efficiency was observed for Faisal canola on BRP-I (10.0%), followed by Aari canola on BRP-II (13.3%), and Nifa Gold on BRP-III (4.0%). The regeneration efficiency of Aari canola was 1.2-fold and 2.3-fold lower than Westar on BRP-I and BRP-II, respectively, while 1.3-fold higher on BRP-III.

For the total number of shoots, Aari canola produced highest number of shoots on BRP-I and BRP-II with 137 and 164.33 shoots from 50 explants, respectively, while Faisal canola produced the highest number of shoots (129 shoots) on BRP-II (Figure 2b,f,j). Punjab canola produced least number of shoots on BRP-I (14.3 shoots/50 explants) and BRP-III (15.6 shoots) while it was Westar, which showed the lowest response on BRP-II with 24.6 shoots from 50 explants. The average number of shoots per explant was in the range of 0.2–2.7, 0.4–2.5 and 0.3–3.2 on BRP-I, BRP-II and BRP-III, respectively. The number of shoots produced by Aari canola was 1.7-fold, 5.2-fold and 1.0-fold higher than Westar on BRP-I, BRP-II and BRP-III respectively.

Overall, the regeneration conditions provided in BRP-I and BRP-III seemed more conducive for generating shoots from detached petioles for Aari canola and Westar. The regeneration conditions in BRP-II appeared favorable for Faisal canola.

### 2.3. Shoot Regeneration from Hypocotyls

Regeneration from hypocotyl segments usually started after 3 weeks on SIM although callus formation started in the second week. The upper end of the hypocotyl cut 2 mm below the epicotyl region showed more swelling and calli formation, ultimately producing higher number of shoots as compared to the other end of the explant. Shoot regeneration was rarely observed from the middle rib portion.

The highest regeneration efficiency from hypocotyls was observed in Aari canola on BRP-III (50.6%) followed by Westar and Nifa Gold with 37.3% and 32.6%, respectively (Figure 1k). The highest regeneration efficiency on BRP-I and BRP-II was of Nifa Gold with 15.3% and 21.3%, respectively, as compared to the other cultivars, suggesting the suitability of these protocols for Nifa Gold for obtaining regeneration from hypocotyls sections. Faisal canola, Punjab canola and Westar were least responsive on BRP-I, while, Aari canola did not respond to BRP-II at all. When the efficiency of Aari canola was compared with Westar, it was 5.3-fold higher on BRP-I, 17.3-fold lower on BRP-II and 1.3-fold higher on BRP-III.

When the total number of shoots regenerated were counted, it was Nifa Gold that produced highest number of shoots on BRP-I (60.3 shoots from 50 explants) and BRP-II (170 shoots/50 explants) while Aari canola produced highest shoots on BRP-III (121 shoots/50 explants) (Figure 2c,g,k). The hypocotyl segments of Aari canola did not respond to BRP-II at all like Faisal canola and Punjab canola on BRP-I. In contrast, Westar showed regeneration on all three protocols, with highest shoot count on BRP-III (82 shoots/50 explants), followed by BRP-II with 61.6 shoots and BRP-I with 15 shoots. The average number of shoots generated from hypocotyls were in the range of 0–1.2, 0–3.4, and 0.4–2.4 per explant on BRP-I, BRP-II and BRP-III, respectively.

Overall, the regeneration conditions of BRP-III appeared more responsive to hypocotyls of three cultivars, Aari canola, Nifa Gold and Westar producing more than one shoot per explant. BRP-I and BRP-II protocols were found suitable for regeneration from hypocotyls of Nifa Gold only. The conditions in BRP-I were not suitable for obtaining plants from hypocotyls of any cultivars other than Nifa Gold. Likewise, the conditions in BRP-II did not appear suitable for hypocotyls of Aari canola, Faisal canola and Punjab canola too. However, hypocotyl explants of Nifa Gold and Aari canola generated good number of shoots on BRP-II and BRP-III, respectively, though they were far less efficient and slower than the cotyledonary explants.

### 2.4. Shoot Regeneration from Roots

Roots explants turned green and showed extraordinary elongation on SIM followed by little swelling and callus formation in the previous 2–3 weeks but could not form any shoots till the fourth week on the medium. The conditions in BRP-I and BRP-II were not suitable for regeneration from roots for any cultivar. Regeneration was observed only on BRP-III, with Westar showing the highest regeneration efficiency (42.66%) followed by Punjab canola with 19.3% regeneration efficiency (Figure 1d,h,l). The total number of shoots from these two cultivars was 135 and 26 shoots from 50 explants for Westar and Punjab canola, respectively (Figure 2d,h,l). A significantly small number of shoot formations was observed in Aari canola and Nifa Gold, whereas, Faisal canola could not regenerate any shoots at all.

**Figure 3:**
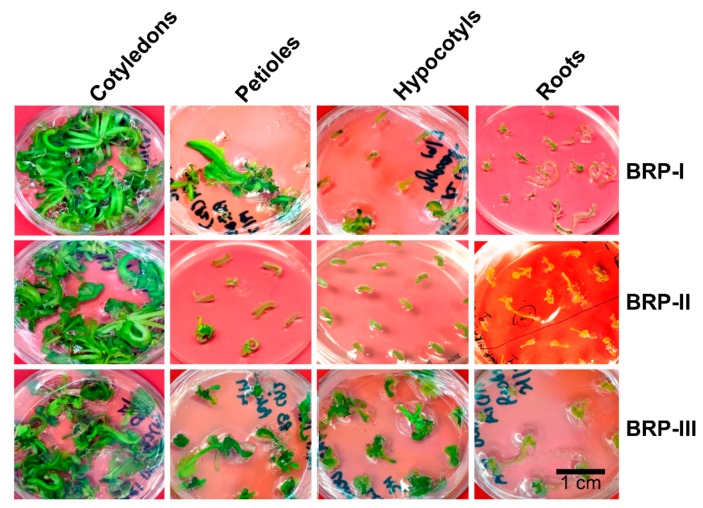
Regeneration of Aari canola on different regeneration protocols from four different explants cotyledons, excised petioles, hypocotyls and roots. Only representative figures of the three replicates are shown. Pictures were taken after 30 days. Abbreviations: BRP, Brassica regeneration protocol.

Figure 3 shows the regeneration of Aari canola from four different explants on different protocols.

### 2.5. Effect of Explant, Regeneration Conditions, and Their Interaction on In Vitro Regeneration

The data obtained was analyzed for the significance and the degree of effect of the explant type, the growth regimes, as well as their interaction on the regeneration of all the cultivars. A standard analysis of variance was applied to analyze the regeneration data. Table 1 shows the summary of the analyses. The explant had a highly significant effect on regeneration (*p* ≤ 0.0001). The effect of regeneration conditions was less significant in Westar compared to other cultivars (*p* ≤ 0.001), whereas, it was non-significant in Nifa Gold. Only in one cultivar, Punjab canola, it was highly significant (*p* ≤ 0.0001). The effect of ‘explant × regeneration conditions’ was statistically non-significant only in one cultivar, Nifa Gold (*p* ≥ 0.05), whereas it was less significant for Westar compared to the other three. The effect of replication was non-significant on all cultivars except Punjab canola.

In terms of percentage contribution of these effects to the regeneration efficiency, the effect of explant type was highest from all the other factors except for Westar (Table 2). It was highest in Aari canola (91.87%) while lowest in Westar (24.13%). The effect of regeneration conditions was much more pronounced on Westar regeneration (57.21%) compared to that of the commercial varieties. The regeneration conditions were less likely to influence the regeneration of Aari canola and Nifa Gold unlike of Faisal canola and Westar. The effect of ‘explant × regeneration conditions’ was variable among different cultivars. It was highest for Faisal canola (27.12%) and Punjab canola (31.44%) while lowest for Aari canola (4.95%). The contribution of the replicate effect was negligible. The lower residual error values of these cultivars except Nifa Gold indicate a high degree of uniformity in their regeneration response.

### 2.6. Determining Kanamycin Sensitivity Levels

From the regeneration experiments, cotyledons were found to exhibit highest regeneration potential on all three protocols. Likewise, the regeneration protocol, BRP-I, was observed to be more conducive for multiple shoot regeneration compared to the other two protocols used in this study. Therefore, in this experiment, only cotyledons were used to determine sensitivity of each cultivar to kanamycin on BRP-I at 0, 10, 15, 20, 25 and 30 mg/L of kanamycin. In total, 30 explants (10 explants per plate) were divided into three subgroups. The sensitivity level of each cultivar was determined where explants did not produce any viable shoots.

**Figure 4:**
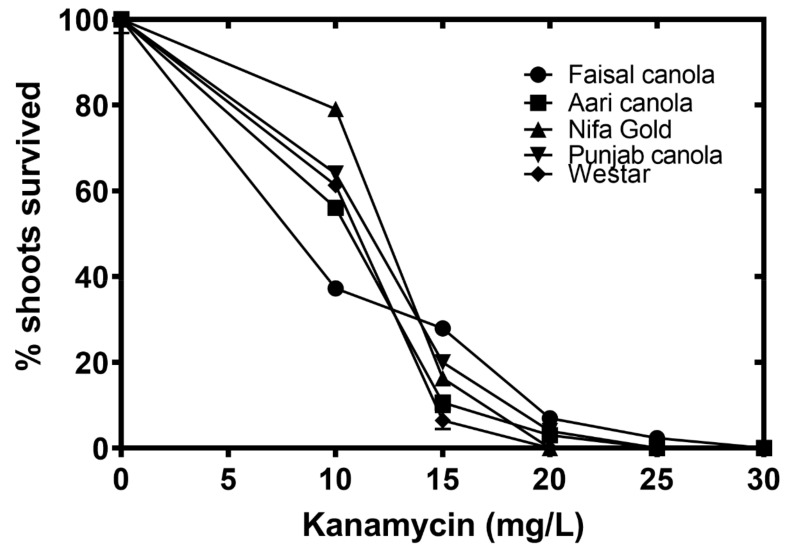
Determining optimum concentration of kanamycin. Cotyledons of 4 day old seedlings of Faisal canola (circles), Aari canola (squares), Nifa Gold (triangles), Punjab canola (inverted triangles) and Westar (diamonds) were cultured on different concentrations of kanamycin. Data points represent the means ± SE of three replicates.

Different cultivars showed a mixed response to kanamycin dosage. The minimum concentration at which complete inhibition was observed was different for each cultivar. For example, it was 20 mg/L for Westar and Nifa Gold, 25 mg/L for Punjab and Aari canola and 30 mg/L for Faisal canola (Figure 4).

### 2.7. Completion of Regeneration Cycle for Aari canola

**Figure 5:**
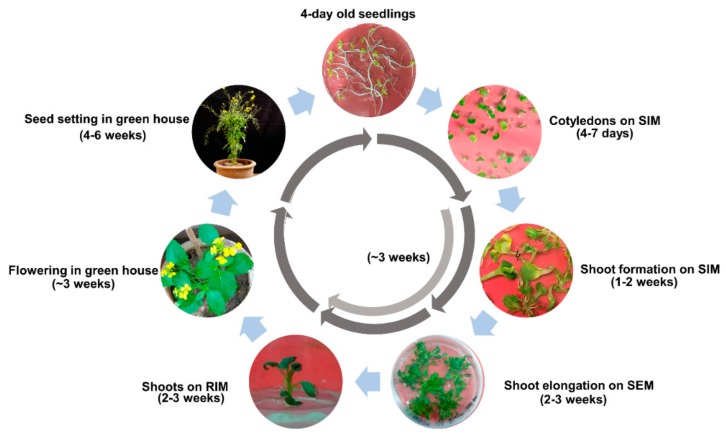
Complete regeneration cycle of Aari canola on BRP-I. Different phases of plant regeneration along with the timeline are shown. The regeneration cycle from cotyledons to seed collection takes ~18 weeks. It can be shortened by another two weeks by skipping the SEM step and increasing the SIM incubation for three weeks (light arrow). Abbreviations: SIM, shoot initiation medium; SEM, shoot elongation medium; RIM, root induction medium.

The Aari canola shoots were matured for flowering and seed setting, as described in the Section 4.5 of the Methods. The regeneration cycle with timescale for recovering fully mature plants is shown in Figure 5. It was observed that the complete recovery cycle from cotyledons would take ~18 weeks, around two week earlier than *Brassica* oleracea, which has been reported to take ~20 weeks on the same protocol and same explant [23].

### 2.8. Rooting Efficiency

Almost all isolated shoots were capable of developing roots successfully regardless of the explant type they originated from (data not shown).

## 3. Discussion

The present study was carried out to evaluate the regeneration potential of commercial cultivars of *Brassica* belonging to *Brassica juncea* and *Brassica napus*. The regeneration and transformation protocols for *Brassica* are mainly limited to model cultivar Westar and attempts have been made to extend these developments to transform the locally adapted *Brassica* cultivars in many parts of the world [28,29,30,31,32]. Although the success of genetic transformation depends on several factors including susceptibility of the genotype to *Agrobacterium* infection, explant type as well as age, and in vitro regeneration efficiency are the key factors. Transformation of the elite varieties is manly hindered because of the high degree of recalcitrance of these varieties to regeneration protocols [23]. Therefore, the first pre-requisite to embark on transformation experiment of elite cultivars is to evaluate their regeneration potential to available regeneration protocols.

Regeneration in *Brassica* has been reported from several tissues and organs including leaves, stem sections, petioles, roots, hypocotyls, cotyledons, immature zygotic embryos, protoplasts and cell suspension cultures [16,27,33,34,35,36,37,38,39,40,41]. Out of these, cotyledons, hypocotyls and roots have been frequently used for genetic transformation [2,16,19,37,42,43]. Therefore, only the commonly used explants were tested in this study. Explant age and type also significantly influences regeneration potential. Ono, Takahata and Kaizuma [42] compared regeneration from the explants isolated from seedlings of different ages. The study reported that explants from 4 day old seedlings gave higher regeneration response compared to the those isolated from 5 and 6 day old seedlings. In another study, when the explant age was increased from 4 to 10 days, the regeneration frequency was significantly decreased [44]. Therefore, in this study, only 4 day old seedlings were used for explant preparation.

Cotyledonary explants showed the highest regeneration efficiency on all the tested protocols followed by petioles and hypocotyls, respectively (Figure 1). Overall, the roots were found to be least responsive among all the types of explants used (Figure 1d,h,l). This observation was in line with earlier studies. Zhang and Bhalla [34] tested the regeneration potential of seven Australian commercial cultivars of *Brassica napus* using cotyledons, hypocotyls, and roots. In six cultivars, the regeneration response from roots was comparatively low and slow compared to cotyledons and hypocotyls.

We observed that the regeneration protocol BRP-I was the most conducive for shoot formation from cotyledons (up to 10.9 average shoots per explant) while BRP-II was best for petioles (up to 3.2 average shoots per explant) and BRP-III promoted shoot formation both in hypocotyls (up to 3.4 average shoots per explant) and root explants (up to 2.7 shoots per explant) (Figure 2). We applied these protocols without introducing any major modification. The regeneration of these cultivars, including that of Aari canola, could be further improved upon by optimizing the plant regeneration conditions. It has been observed that varying the media components affects the shoot formation efficiencies significantly [33,35,36,37,42,43,45,46,47]. The fact that huge variations have been observed among different explants, a generalized recommendation cannot be made. For example, all the three protocols can be efficiently used to recover transformants of Aari canola from cotyledons, while the regeneration efficiency of other explants was greatly reduced in these protocols (Figure 1). The poor regeneration of elite varieties except Aari canola indicates the recalcitrant nature of these cultivars to regeneration as reported in the literature. The regeneration response of Westar to BRP-III from all the explants confirms the fact that it was developed for Westar (Figure 1 and Figure 2). Nevertheless, this study provides a snapshot of the in vitro regeneration potential of these cultivars on three different regeneration protocols.

In vitro regeneration in the genus *Brassica* is highly genotype specific and huge variations have been reported in the regeneration potential of different genotypes. In the present study, all the tested genotypes exhibited variable regeneration response in a manner that appears highly genotype specific. For example, huge variations were observed in regeneration from different explants ranging from 6% to 73%, 4% to 79.3%, 0% to 50.6% and 0% to 42.6% from cotyledons, petioles, hypocotyls and roots, respectively (Figure 1). Similar variations have been reported in *Brassica*. Hachey, et al. [48] screened six cultivars of *Brassica campestris* and observed 0–70% regeneration in these genotypes. Ono, Takahata and Kaizuma [42] investigated the regeneration potential of 100 genotypes of *Brassica napus* by in vitro regeneration and reported huge variations in the regeneration response, which ranged from no regeneration at all to 97%. Zhang and Bhalla [34] reported huge variations (0–96.7%) in seven Australian cultivars of *Brassica napus*. Similar genotype-dependent variations among different *Brassica* species have been reported in several studies [23,33,35,36,37,38,39,40,43,45,46,47,49].

The regeneration response appeared specific to the type of cultivar and media components used. For example, Aari canola showed exceptional regeneration on all the tested regeneration protocols. The model variety, Westar, showed a good regeneration on BRP-III but lagged far behind Aari canola. Both Faisal and Punjab canola were least responsive to BRP-I and BRP-III. The results presented here show that explant type had a significant effect on the in vitro regeneration response of the tested cultivars (Table 1 and Table 2). The effect that regeneration conditions provided was less significant compared to the explant type and often non-significant like that of the replicate. Khehra and Mathias [27] studied the effect of genotype along with explant type on shoot regeneration frequency in four *Brassica napus* varieties, one spring (Westar) and three winter (Ariana, Cobra and Libravo) by culturing cotyledons, hypocotyls and stem sections on different growth mediums. The study showed that both the genotype and explant type had significant effect on shoot regeneration frequency.

Determination of the threshold values of the selective agent, kanamycin in this study, is a preliminary requirement before starting transformation experiments that will allow the selective growth of transformants while killing the non-transformants. Different cultivars showed a complete inhibition at different kanamycin concentrations (Figure 4) perhaps due to the genotypic variations. The findings of this study are in close agreement with [50] who reported that the optimum kanamycin concentration for mustard (*Brassica juncea* Coss.) was 30 mg/L.

A notable observation of this study is the exceptional regeneration response of an elite variety Aari canola to different regeneration conditions particularly from cotyledons. The regeneration efficiency of Aari canola from these explants was found to be up to 7.5-fold higher than the model cultivar Westar (Figure 1a,e,i), whereas, when compared in terms of total number of shoots obtained per explant, it was up to 21.9-fold higher than Westar (Figure 2a,e,i). Shoots of Aari canola were successfully grown to maturity using the procedure outlined in BRP-I [23]. The complete regeneration of mature Aari canola plants took approximately 18 weeks (Figure 5), which is 2 weeks shorter than the *Brassica oleracea* for which this protocol was originally designed [23]. The efficient regeneration of Aari canola both in terms of regeneration efficiency and as well as total number of shoots obtained, and relatively quicker recovery of mature plants make it a good candidate for genetic transformation. One of the reasons of faster recovery of mature plants grown from in vitro regenerated shoots may be since Aari canola is a short-duration variety. However, such a correlation between the cropping type, short-day or long-day, with the in vitro regeneration potential has not been observed in *Brassica* [42]. Aari canola is a recently approved, high-yielding commercial canola cultivar with many good agronomic attributes, and is currently grown in many parts of the country [51]. Transformation of an elite variety has several advantages over the transformation in a model genotype. Transformations are usually made in a few selected genotypes of a species, which can be easily manipulated by genetic means. Often, these genotypes have poor agronomic performance and therefore, the engineered traits must be transferred into commercial yet cross-compatible varieties through crossing. After crossing, it takes several more years breeding work, labor and cost to recover the recurrent parent genome, and to minimize the ‘linkage drag’. The associated labor and costs with the back-crossing programs increase several times if the number of traits to be transferred increases [10,52]. Aari canola belongs to *Brassica juncea* (Table 1), which is the most widely cultivated genotype in the Southeast Asian countries like India and Pakistan [30]. The use of Aari canola (commercial variety) in transformation experiments will help expedite the improvement of canola through genetic transformation against biotic and abiotic stresses as well as facilitate the functional genomic studies.

In conclusion, we have used simple regeneration protocols, with slight modifications, to successfully regenerate shoots from different explants of commercial *Brassica* varieties. Our work has identified commercial cultivar, Aari canola, which is highly responsive to regeneration conditions tested. The quicker recovery of in vitro plants and extra-ordinary regeneration potential make this variety an ideal candidate for future transformation-based studies. The regeneration conditions and the kanamycin levels identified in this work will be useful for improving canola through different genetic engineering approaches against various biotic and abiotic stresses as well as functional genomic studies.

## 4. Materials and Methods

### 4.1. Plant Material and Growth Conditions

Five different cultivars (four local and one model) belonging to *Brassica napus* and *B. juncea* were obtained from different institutes of Pakistan (Table 3).

### 4.2. Sterilization and Sowing

Seeds were stratified at 4 °C for 48 h before sowing to ensure uniform germination. Seeds were surface sterilized by immersing in 100% ethanol for 2 min followed by immersing in commercial bleach (sodium hypochlorite solution) containing 4–6% available chlorine plus 2–3 drops of 10% tween-20 for 10 min. The seeds were then rinsed four times with sterile distilled water in a laminar flow and placed on sterile filter paper to dry and germinated on seed germination media (MS salts, 3% sucrose, 1 mg/L pyridoxine, 1 mg/L nicotinic acid, 10 mg/L thiamine-HCl, 100 mg/L myo-inositol, 4 g/L phytagel, pH 5.7–5.8) at a density of 20 seeds per 90 × 30 mm petri dish in a growth room maintained at 23 °C at 16 h light/8 h dark cycles under a light intensity of 50 µmol m^−2^ s^−1^ provided by cool fluorescent bulbs.

### 4.3. Explant Isolation and Shoot Induction

Four different explants intact cotyledons with approximately 2 mm of petiole, hypocotyls (approximately 3–4 mm), petioles (approximately 2 mm, with the ‘leaf of the cotyledon removed’, and roots (3–4 mm) sections were subjected to in vitro regeneration conditions. Four day old seedlings were used for explant isolation. A pair of long, sterile forceps were used to remove the seedlings from the germination media and placed in sterile petri dishes. Explants were cut and separated using a sharp scalpel blade and transferred to either shoot induction media (SIM) or callus induction media (CIM) (see Table 4 for composition). Cotyledons were excised with a sharp scalpel blade with 2 mm petiole avoiding any part of meristem. Explants were placed on the medium in such a way that the petiole was embedded in media and the cotyledonary lamella was clear of the media. 10 explants were established on each plate and transferred to a growth room at 23 °C under scattered light. A total of 50 explants of all varieties were used for each experiment, and the experiments were repeated three times. Cultures were transferred to fresh medium every two weeks. The explants were subjected to regeneration under three different regeneration protocols termed as *Brassica* Regeneration Protocols, BRP-I [23], BRP-II [53] and BRP-III [19]. A number of protocols for the in vitro regenerations of different *Brassica* genotypes have been reported in the literature, which are highly specialized for a certain genotype and are often complicated [1,3,16,28,29,32,33,34,35,36,37,40,42,43,44,45,46,48,54,55,56,57,58,59,60]. Further, different explant types have been shown to respond differently to different hormonal combinations [44], which is further complicated by the fact that regeneration in *Brassica* is highly variable among different genotypes [42]. These protocols were chosen because of the simplicity and fewer steps involved, which ultimately will enable an easier transfer of the protocols to other laboratories, as well as the nature of work in our lab.

The final data was recorded in two ways: (a) total number of explants showing regeneration and (b) the total number of shoots regenerated from all the number of explants. The first type of data did not consider the multiple number of shoots per explant, therefore, we chose to record data for the total number of shoots to account for the fact that many explants regenerated multiple shoots.

### 4.4. Kanamycin Selection Levels

Kanamycin was added in the shoot induction medium (SIM) (see Table 4 for composition) at a concentration of 0, 10, 15, 20, 25 and 30 mg/L. The cotyledons were obtained, excised and placed on SIM as described above for regeneration using BRP-I. Culture conditions and sub-culturing duration were also the same. Cultures were monitored regularly, and the time taken for shoot induction at different levels of kanamycin noted, together with the total number of shoots per explant after two-week intervals.

### 4.5. Growing In Vitro Regenerated Shoots to Maturity

The selected in vitro regenerated shoots of Aari canola were grown to maturity for flowering and seed setting as described in Hundleby and Irwin [23] with slight modifications to determine the timescale for the complete recovery of mature plants. Briefly, shoots were isolated and transferred to 100 mL jars containing 25 mL root induction medium (RIM; ½ MS salts, 1 mL vitamin B_5_ stock, 1% (*w*/*v*) glucose, 4 g/L phytagel and 2 mg/L IBA). The sub-culturing was repeated in the case of multiple shoots arising from a single explant until single-stemmed shoots were obtained. The shoots were maintained at 40 µmol m^−2^ sec^−1^ 16 h/8 h day/night cycle at 23 °C until enough root mass was obtained. The rooted shoots were transferred to sterile peat pots before transferring them to a greenhouse. Plants were transferred to pots filled with compost-soil mixture. The pots were covered with white polythene bags to help plants adjust to the greenhouse conditions. The plants were grown at 18/12 °C day/night temperature and 16/8 h day/night photoperiod under ambient light conditions (~200 µmol m^−2^ sec^−1^). Plants were fed weekly with 2:1:1 NPK solution. Once the plants were established under greenhouse conditions, the white bags were gradually removed. After seed setting and pod formation, the watering schedule was reduced to help pods dry on the plant. Pods were harvested when pods were 70–80% brown in color to avoid seed loss from pod shattering. The collected pods were dried for 2–3 days to eliminate moisture, and then stored.

### 4.6. Rooting Efficiency

The rooting efficiency of regenerated shoots from each protocol was determined by taking five (05) random shoots from each replicate by transferring them to RIM (see Table 4 for composition). Two of the rooted shoots were transferred to peat moss and grown under greenhouse conditions for seed setting as described in Section 4.5.

### 4.7. Statistical Analysis

The statistical significance of the data obtained was validated using Chi-square test at *p* = 0.05 with 95% confidence interval (CI) and a two-way ANOVA at α = 0.05 by using the statistical software SPSS v18.

## Figures and Tables

**Table 1:** Mean square and significance levels from analysis of variance of data from the regeneration of five *Brassica* cultivars.

Source of Variation	DF	Faisal Canola	Aari Canola	Nifa Gold	Punjab Canola	Westar
Explant (E)	3	6552 ****	368,487 ****	26,280 ***	6644 ****	10,035 ****
Regeneration Conditions (RC)	2	8297 ***	11,116 ***	1026 ^NS^	8261 ****	35,692 **
Interaction (E x RC)	6	2540 ****	9938 ****	1762 ^NS^	3202 ****	2323 **
Replicate	8	115.9 ^NS^	795.6 ^NS^	891.1 ^NS^	318.7 ***	302.9 ^NS^
Residual Error	16	236.1	596.7	769.1	180.1	432.5

*, **, ***, **** Significant at *p* = 0.05, 0.01, 0.001 and 0.0001, respectively; NS, non-significant.

**Table 2:** Percent contribution of explant type, growth conditions, and their interaction on the regeneration frequencies of five *Brassica* cultivars.

Source of Variation	Percent Contribution of a Treatment to the Total Experimental Variations				
	Faisal canola	Aari canola	Nifa Gold	Punjab canola	Westar
Explant Type (E)	34.98	91.87	71.09	32.62	24.13
Regeneration Conditions (RC)	29.53	1.848	1.850	27.04	57.21
Interaction (E x RC)	27.12	4.956	9.533	31.44	11.17
Replicate	1.650	0.529	6.428	4.173	1.942
Residual Error	6.720	0.797	11.09	4.727	5.548
Total	100.0	100.0	100.0	100.0	100.0

**Table 3:** List of cultivars with their sources used in this study.

Sr. No.	Cultivar	Species	Source
1	Aari canola	*Brassica juncea*	Oilseed Research Institute, Ayyub Agriculture Research Institute (AARI), Faisalabad, Pakistan
2	Faisal canola	*Brassica napus*	Oilseed Research Institute, Ayyub Agriculture Research Institute (AARI), Faisalabad, Pakistan
3	Punjab canola	*Brassica napus*	Oilseed Research Institute, Ayyub Agriculture Research Institute (AARI), Faisalabad, Pakistan
4	Nifa Gold	*Brassica napus*	Nuclear Institute for Food and Agriculture, Peshawar, Pakistan
5	Westar	*Brassica napus*	National Oilseed Development Program (NODP), National Agricultural Research Centre (NARC), Islamabad, Pakistan

**Table 4:** Composition of different mediums used in the study.

Reagents	BRP-I		BRP-II			BRP-III			
	SIM	GRM	CIM	SIM	RIM	CIM	SIM	SOM	RIM
MS salts (mg/L)	4.43		4.43	4.43	4.43	4.43	4.43	4.43	2.215
Gamborg’s salts (mg/L)		3.1							
Sucrose (mg/L)	30	10	30	30	30	20	20	20	10
Vitamin Stock (ml/L)	1	1	1	1	1	1	1		
Phytagel (g/L)	4	4	4	4	4	4	4	4	4
BAP (mg/L)	2		6	1		0.75	3	0.00125	
NAA (mg/L)					0.1	0.2	0.2		
IBA (mg/L)									1
AgNO_3_ (mg/L)						5	5		
GA_3_ (mg/L)						0.01	0.01		
CaCl_2_ (mg/L)						435	435	435	
KI (mg/L)						0.75	0.75	0.75	0.375
Adenine hemisulfate (mg/L)								40	
PVP 40,000 (mg/L)								500	

BRP, *Brassica* regeneration protocol; SIM, shoot induction medium; GRM, Gamborg’s rooting medium; CIM, Callus induction medium; RIM, Root induction medium; SOM, Shoot outgrowth medium.
